# Colorectal and emergency surgical patients in the literature supporting EHS and AHS guidelines on abdominal wound closure: a granular analysis

**DOI:** 10.1007/s10029-025-03493-7

**Published:** 2025-10-22

**Authors:** Vittoria Bellato, Sue Blackwell, Muhammed Elhadi, Thomas Grove, Lisa Massey, Barbara Vieira, Andreas Denys, Francesco Pata, Gabrielle H. van Ramshorst, 2024 European Society of Coloproctology (ESCP) Collaborating Group, Thomas Pinkney, Erman Aytac, Pamela Buchwald, Niki Christou, Dragomir Dardanov, Alaa El-Hussuna, Nir Horesh, Karoline Horisberger, Per Johansson, James Keatley, Yurij Kosir, Hans Lederhuber, Dion Morton, Mostafa Shalaby, Carolynne Vaizey, Patricia Tejedor, Sanjay Chaudhri, Sharfuddin Chowdury, Audrius Dulskas, Caterina Foppa, Matteo Frasson, Gaetano Gallo, James Glasbey, Michael Kelly, Elizabeth Li, Ana Maria Minaya-Bravo, Peter Neary, Ionut Negoi, Gianluca Pellino, Shaji Sebastion, Beatriz Silva Mendes, Baljit Singh, Aya Riad, Niels-Derrek Schmitz, Kerstin Spychaj, Celine Riess, Liza Ovington

**Affiliations:** 1 Department of Minimally Invasive Surgery, University Hospital of Rome Tor Vergata, Rome, Italy; 2 Institute of Applied Health Research, University of Birmingham, Birmingham, UK; 3 Faculty of Medicine, University of Tripoli, Tripoli, Libya; 4 Department of General and Colorectal Surgery, East Surrey Hospital, Imperial College London, London, UK; 5 Department of Colorectal Surgery, Nottingham University Hospitals, Nottingham, UK; 6 Department of General Surgery, Hospital de Santo Espírito da ilha Terceira, Azores, Portugal; 7 Department of Gastrointestinal Surgery, Ghent University Hospital, Ghent, Belgium; 8 Department of Human Structure and Repair, Ghent University, Ghent, Belgium; 9 Department of Pharmacy, Health and Nutritional Sciences, University of Calabria, Rende, Italy

**Keywords:** Abdominal wound closure, Colorectal; Emergency, Guidelines, Supporting literature, EHS, AHS, European, American

## Abstract

**Background:**

To critically appraise the evidence supporting the European and American Hernia Society (EHS/AHS) guidelines on abdominal wall closure in colorectal and emergency surgery patients, we conducted a granular analysis focused on the representation and quality of available data.

**Methods:**

References that addressed key questions (KQ) and recommendations in the original guidelines were screened and included if colorectal/emergency surgery was reported. Data extraction was performed with a standardised form and authors were contacted for missing data. Quality and risk of bias were assessed independently by two reviewers.

**Results:**

Out of 33 studies included: 15 systematic reviews and one literature review were rated low or critically low (AMSTAR 2); 12 randomised controlled trials had moderate to high risk of bias (Cochrane RoB-2 tool); five observational studies were of low to very low quality (GRADE) with serious risk of bias (ROBINS-I). 32 studies included colorectal (*n* = 15,856) and 14 emergency patients (*n* = 4,582). To answer KQ1 on ‘minimally invasive or open surgery’ and ‘type of incision’ 9/10 studies included colorectal and no studies included emergency patients. 1/4 and 8/9 studies included colorectal/emergency patients for recommendations regarding trocar sites closure (KQ2) and closure after laparotomy (KQ3). Regarding the use of mesh (KQ5), 11/11 studies included colorectal/emergency patients. 3/3 and 1 studies included colorectal patients for KQ6 (abdominal binders) and one for KQ7 (restriction of activity), but none included emergency patients.

**Conclusion:**

Recommendations for KQs 1, 3, 5, 6, and 7 appear applicable to colorectal patients, whereas evidence for KQ2 remains insufficient. For emergency patients, recommendations related to KQs 1, 2, 6, and 7 should not be extrapolated. This study further highlights critical limitations in the evidence base, including the lack of patient-centred outcomes, and underscores the need for targeted, high-quality research in these populations.

**Supplementary Information:**

The online version contains supplementary material available at 10.1007/s10029-025-03493-7.

## Introduction

Incisional hernia (IH) is a common complication following abdominal surgery with an estimated rate of 5–20% [1], that may increase further in some subgroups of patients. With around 400,000 operations performed each year, IH repair is one of the five most common operations of general surgery in the United States [2, 3]. IH may significatively affect the patient quality of life and represents a significant burden for healthcare systems due to costs related to the hernia repair and associated morbidity, with recurrence rates of up to 45% [4]. Recent estimates suggest that a yearly cost reduction of $17 million could be achieved for each 1% decrease in IH operations in the US [5].

Colorectal surgery patients, especially those undergoing cancer resections, are considered at high risk for developing IH. The HART trial reported IH rates of up to 31.8% in patients after elective colorectal cancer surgery at two-year follow-up [7]. In a retrospective population-based study from France [1] including 431,619 patients, colorectal resections accounted for over half of all subsequent incisional hernia repairs. Emergency surgery, which often entails unfavorable conditions for wound healing, has also been associated with increased IH rates, though some studies question its role as an independent risk factor [11, 12].

Despite the growing awareness of this issue, significant heterogeneity persists across studies in terms of both clinical and patient-reported outcomes [6]. A 2021 ESCP survey of 561 colorectal surgeons and trainees further highlighted this variation in practice. Respondents often applied identical abdominal wall closure techniques in disparate clinical scenarios, underscoring the lack of consensus and the need for more risk-adapted, evidence-based guidance [8].

With the aim to propose evidence-based mitigation strategies in reducing the incidence of incisional hernia after abdominal surgery, the European Hernia Society (EHS) published its guidelines on closure of abdominal wall incisions in 2015 [9], which were recently updated in synergy with the American Hernia Society (AHS) in 2022 [10]. However, in the attempt to generalise these recommendations and maximise the evidence available, there was no specific focus on the type of surgery with the literature search including papers reporting on any type of open abdominal surgery.

The current study aims to critically evaluate the evidence cited in the EHS/AHS guidelines with a focus on colorectal and emergency surgery patients. Our goal is not only to assess the representation of these groups in the evidence base but also to reflect on how applicable and robust the recommendations are for these higher-risk populations. We also aim to identify gaps in data that could inform future research.

## Methods

### Study design

This study is a structured granular review aiming to critically appraise the evidence base underpinning the 2022 European Hernia Society (EHS) and American Hernia Society (AHS) guidelines on abdominal wall closure, specifically focusing on colorectal and emergency surgery patients [10].

#### Data sources and search strategy

We examined all references cited in the 2022 EHS/AHS guidelines, which were identified through a systematic literature search conducted by the guideline committee in January 2022 across MEDLINE, Embase, and Cochrane CENTRAL databases. The search encompassed studies on abdominal wall closure techniques without restrictions on surgical indication or urgency.

### Eligibility criteri

Eligibility: A total of 39 references cited as evidence for the updated EHS/AHS guideline recommendations, including the original 2015 guidelines, were screened for inclusion. Studies were considered eligible if they were referenced to answer the guideline key questions (KQs) (Table 4). Of these, 38 met the inclusion criteria: 19 systematic reviews, 13 randomized controlled trials, 1 literature review, and 5 observational studies. Full-text publications were used for data extraction. When complete datasets were not available, corresponding authors were contacted to request raw data. A reminder was sent after two weeks, and if no reply was received within an additional two weeks, the request was documented as unanswered. For each included study, data were collected using a standardized extraction form (Appendix). When colorectal and/or emergency surgery patients were included, subgroup data were recorded. Extracted information included study design, patient numbers, surgical approach, and type of incision. For the colorectal subgroup, additional details were collected when available, such as surgical indication and the use of prophylactic (parastomal) mesh. Data on interventions, such as incision type, closure technique (e.g., small bites vs. mass closure, continuous vs. interrupted suture, suture material), use of prophylactic mesh, abdominal binders, and postoperative activity restrictions, were also recorded.

The primary outcome of interest was the rate of incisional hernia.

### Quality and risk of bias assessment

Risk of bias assessments were performed independently by reviewers in duplicate with conflicts settled by consensus (VB, SB, AD, TG, GvR). Systematic reviews and meta-analyses were assessed independently by two reviewers (ME, LM) using the AMSTAR 2 tool [13], randomised controlled trials were assessed using the Cochrane Risk of Bias RoB2 tool [14] and non-randomised studies using the ROBINS-I tool [15]. GRADE was used to assess the quality of the non-randomised studies [16]. Our analysis focused on interpreting the methodological quality of the systematic reviews by assessing adherence to the domains outlined in AMSTAR 2. Descriptive analyses were performed where and when applicable. P-values < 0.05 were considered statistically significant.

## Results

### Study selection and general characteristics

Out of the 94 references included in the updated guidelines [10], 39 articles were used to support the recommendations and were therefore considered eligible for analysis. These studies encompassed a cumulative sample of 133,460 patients (*n* = 37, as one article [17] did not report the number of patients, and the original guidelines [9] were excluded from this sample) — see Table 1.

**Table 1:** General characteristics of the studies included in the updated guidelines.

	Author	Study Design	Country	Center(s)	*N*. Patients	Funding	*N*. colorectal patients (%)	*N*. emergency patients	(%)		
KQ1** -** Open and minimally invasive abdominal surgery											
18	Kossler-Ebs J.B.	Syst. review/Meta-analysis	Germany	NA	3.490	NA	2.243 (64.3%)	N/A			
19	Petersson J.	RCT	Netherlands	MC	1.044	Swedish Ca Society, Ethicon	1.044 (100%)	N/A			
20	Taylor G.W.	Observational study	U.K.	MC	411	Medical Research Surgery	411 (100%)	N/A			
21	Pecorelli N.	Observational study	Italy	SC	604	NA	604 (100%)	N/A			
22	Lee L.	RCT	Canada	SC	141	Canadian Association of Surgeons	141 (100%)	N/A			
23	Lee L.	Syst. review/Meta-analysis	Canada	NA	6.314	NA	6.314 (100%)	N/A			
24	Lachapelle C.R.	Observational study	USA	SC	423	NA	423 (100%)	N/A			
25	Widmar M.	Observational study	USA	SC	164	ASCRS	164 (100%)	N/A			
26	Cano-Valderrama C.	Observational study	Spain	SC	225	NA	225 (100%)	N/A			
27	Kulkarni A.A.	Syst. review/Meta-analysis	India	NA	1.036	No funding	N/A	N/A			
KQ2** -** Closure of minimally invasive surgery ports											
28	Gutierrez M.	Literature Review	USA	NA	18.533	NA	Not discriminated	Not discriminated			
29	Karampinis I.	Syst. review/Meta-analysis	Germany	NA	31.516	NA	N/A	N/A			
30	Connell M.B.	Syst. review/Meta-analysis	Canada	NA	3.340	NA	N/A	N/A			
31	Lyu Y.	Syst. review/Meta-analysis	China	NA	5.654	NA	N/A	N/A			
KQ3** -** Closure of laparotomy incisions											
32	Henriksen N.A.	Syst. review/Meta-analysis	Denmark	NA	3.641	No funding	1.895 (52.0%)	Not discriminated			
33	Henriksen N.A.	Syst. review/Meta-analysis	Denmark	NA	10.130	NA	Not discriminated	Not discriminated			
34	Patel S.V.	Syst. review/Meta-analysis	Canada	NA	19.174	No funding	Not discriminated	Not discriminated			
35	Wu X.	Syst. review/Meta-analysis	China	NA	7.458	NA	Not discriminated	Not discriminated			
36	Ademuyiwa A.O.	RCT	Nigeria/UK	MC	5.788	NIHR Global Health	931 (16.1%)	3873 (66.9%)			
37	Ruiz-Tovar J.	RCT	Spain	MC	150	NA	150 (100%)	150 (100%)			
38	Olmez T.	RCT	Turkey	SC	890	No funding	173 (19.4%)	105 (11.8%)			
39	Albertsmeier M.	RCT	Austria	MC	425	Aesculap AG, German Research	242 (56.9%)	N/A			
40	Grat M.	RCT	Poland	SC	268	Poland National Science Centre	N/A	N/A			
KQ5** -** Prophylactic mesh augmentation											
41	Jairam A.P.	Syst. review/Meta-analysis	Netherlands	NA	1.815	NA	Not discriminated	Not discriminated			
42	Tansawet A.	Syst. review/Meta-analysis	Thailand	NA	2.716	NA	469 (17.3%)	Not discriminated			
43	Payne R.	Syst. review/Meta-analysis	U.K.	NA	727	NA	107 (14.7%)	Not discriminated			
44	Ahmed J.	Syst. review/Meta-analysis	Pakistan	NA	3.000	NA	Not discriminated	N/A			
45	Hassan M.A.	Syst. review/Meta-analysis	Canada	NA	916	NA	Not discriminated	Not discriminated			
46*	Timmermans L.	RCT	Netherlands	MC	480	B. Braun Surgical, Baxter Health	162 (33.8%)	N/A			
47	Pizza F.	RCT	Italy	SC	100	No funding	N/A	53 (53%)			
48*	Jairam A.P.	RCT	Netherlands	MC	480	B. Braun Surgical, Baxter Health	162 (33.8%)	N/A			
49	Jakob M.O.	RCT	Switzerland	SC	48	Strattice, Allergan	Not discriminated	48 (100%)			
50	Pizza F.	RCT	Italy	SC	200	No funding	Not discriminated	200 (100%)			
51	Valverde S.	RCT	Spain	MC	332	W.L. Gore and Associate	240 (72.3%)	153 (46.1%)			
KQ6** -** Postoperative care											
52	Rothman J.P.	Syst. review/Meta-analysis	Sweden	NA	578	NA	Not discriminated	N/A			
53	Jiang N.	Syst. review/Meta-analysis	China	NA	968	NA	60 (6.2%)	N/A			
54	Ossola P.	Syst. review/Meta-analysis	Italy	NA	281	NA	98 (34.9%)	N/A			
KQ7** -** Restriction of activity after open abdominal surgery											
55	Loor M.M.	Syst. review/Meta-analysis	USA	NA	0	NA	Not discriminated	N/A			

*NA* Not applicable, *N/A *Not applicable, patients were not included in the studies or it is unclear if those patients were included in the respective study. Not discriminated = when the number of the respective patients is not discriminated, *MC* Multicentric study, *SC* single center *Articles 46 and 48 corresponds to the same RCT (one is follow-up)

Five articles were excluded from our analysis as they did not pertain to colorectal or emergency surgical patients [27, 29–31, 40], in addition to the original guideline article [9].) 33 studies met inclusion criteria and were analysed [26, 28, 32–39, 41–55]. These included 12 randomised controlled trials (RCTs) [19, 22, 36–39, 46–51], 15 systematic reviews/meta-analyses [18, 23, 32–35, 41–45, 52–55], 1 literature review [28] and 5 observational studies [20, 21, 24–26]. Among these studies, 32 included colorectal surgical patients [26, 28, 32–39, 41–46, 48–55] and 16 included patients undergoing emergency surgery [28, 32–38, 41–43, 45, 47, 49–51] - see Tables 2 and 3. Among these, 32 studies included colorectal surgery patients [26, 28, 32–39, 41–46, 48–55], and 16 studies included patients undergoing emergency surgery [28, 32–38, 41–43, 45, 47, 49–51]-see Tables 2 and 3.

**Table 2.**
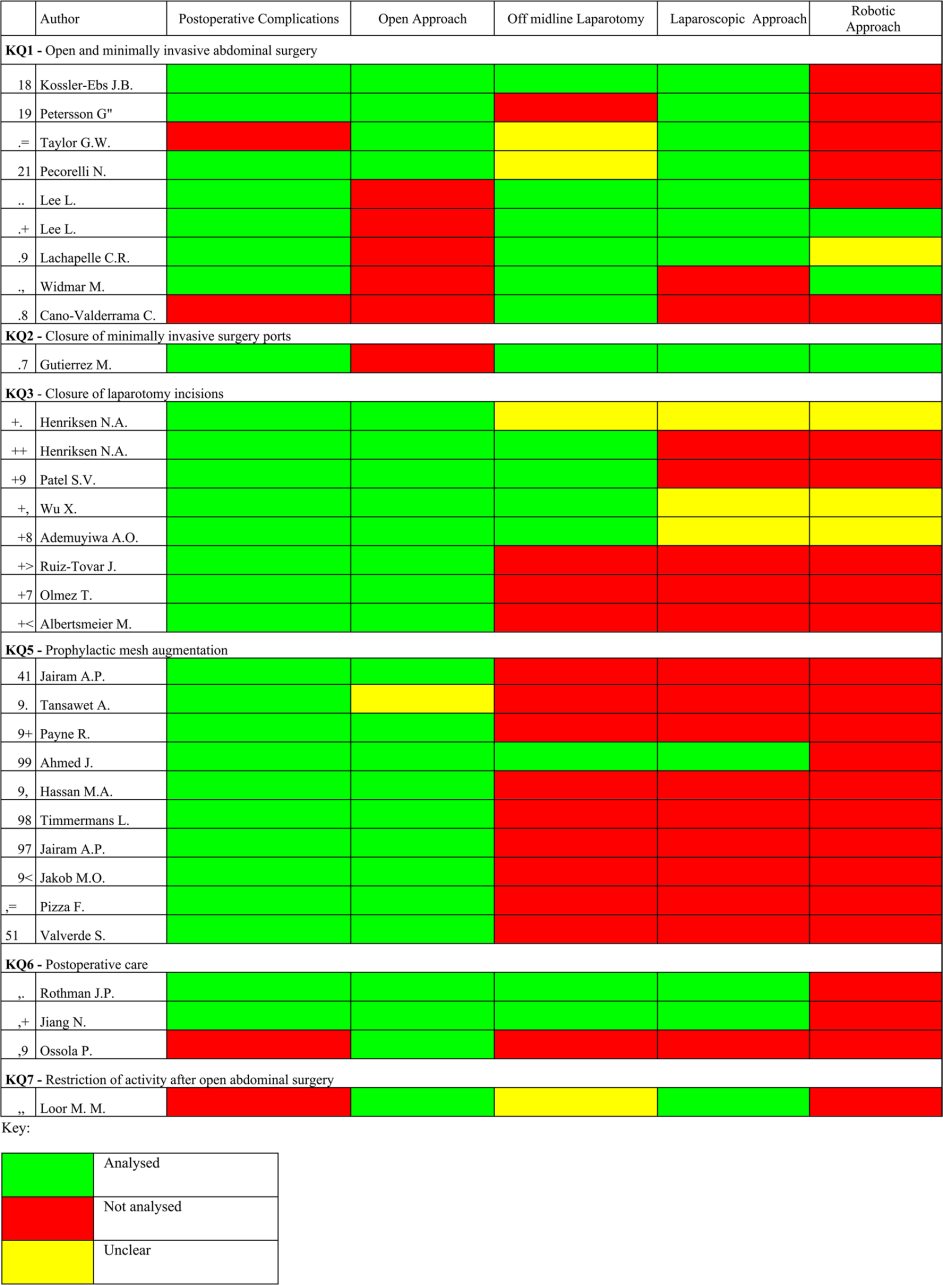
Overview of studies including colorectal patients

**Table 3.**
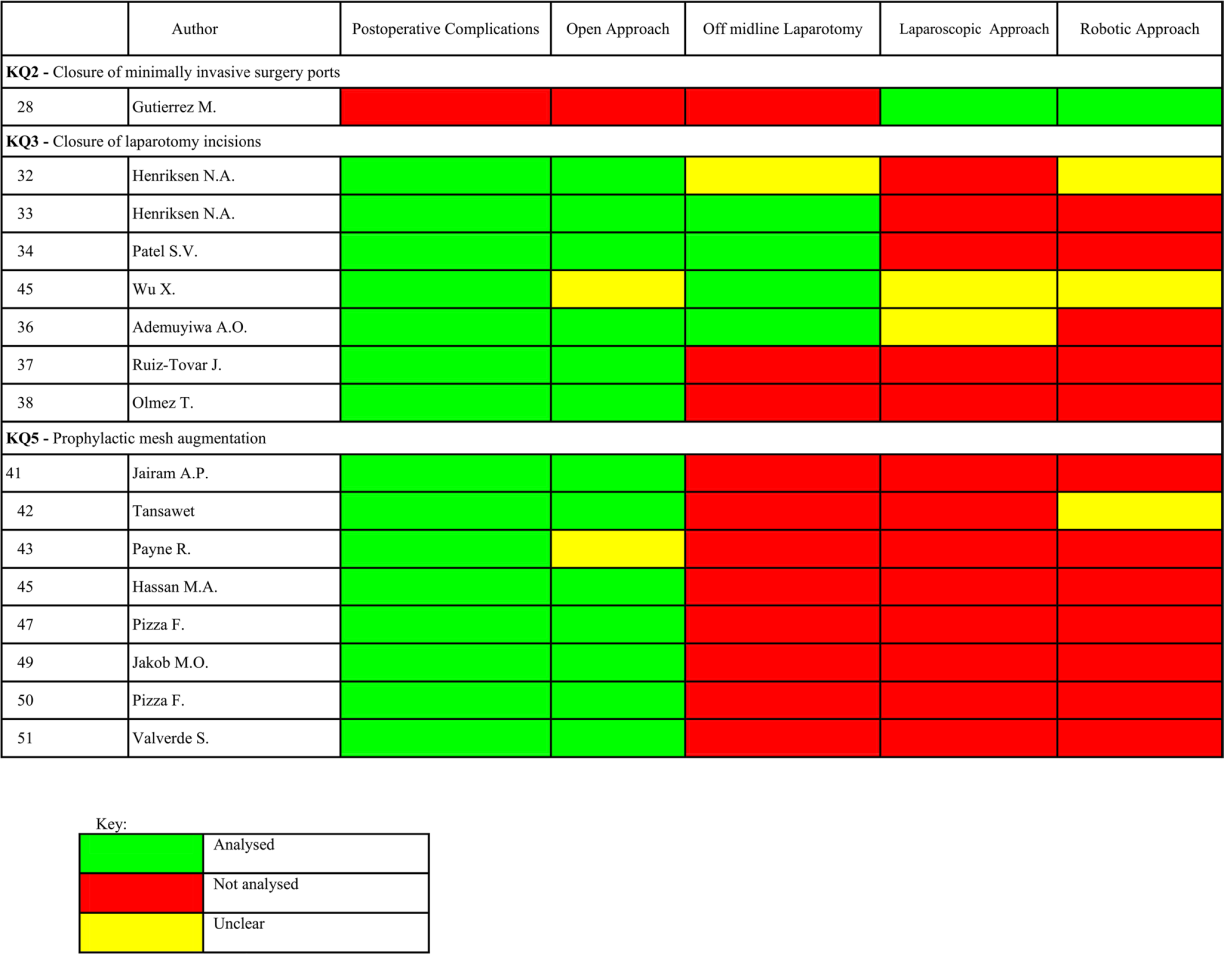
Overview of studies including emergency patients

### Study quality assessment

#### Description of systematic reviews and meta-analyses

Systematic reviews were assessed using the AMSTAR 2 tool [13], which evaluates 16 domains and identifies 7 critical domains to determine confidence in review results. Nearly all systematic reviews were rated as low or critically low in overall confidence. The most common weaknesses were lack of a registered protocol, inadequate reporting of the search strategy, absence of justification for study exclusions, and insufficient consideration of publication bias.

#### Description of randomised control trials (RCTs) 

RCTs were assessed using the Cochrane Risk of Bias 2 (RoB 2) tool [14]. Risk levels (low/moderate/high) were assigned across five domains: randomisation process, deviations from intended intervention, missing outcome data, outcome measurement, and selective reporting. Most RCTs were classified as moderate or high risk of bias, mainly due to missing data, short follow-up, absence/variation of protocol or low statistical power. Outcome definitions varied widely (clinical vs. imaging vs. patient-reported), making comparisons challenging.

#### Description of observational studies

Observational studies were evaluated using ROBINS-I [15] and graded for overall evidence quality using GRADE [16].Four studies [20, 24–26] were rated as very low quality, and one [21] as low quality. All were rated as having serious risk of bias due to confounding, selection bias, or poorly defined outcomes.

## Baseline characteristics of the included studies

### Colorectal surgery patients studies

In total, 32 articles included colorectal patients of which twenty-one discerned the proportions of colorectal patients [18–26, 32, 36–39, 42, 43, 46, 48, 51, 53] and [54]) - Table 1. Of these, nine studies focused exclusively on colorectal patients [19–26] and [37]. Eleven articles did not specify the number of colorectal patients included [28, 33–35, 41, 44, 45, 49, 50, 52] and [55] and the remaining twelve articles included colorectal patients as part of a heterogeneous group of surgical patients [18, 32, 36, 38, 39, 42, 43, 46, 48, 51, 53] and [54] and indicated the proportion.

Regarding the surgical setting, 24 studies included elective colorectal patients [26, 32–35, 38, 39, 43–48, 51, 53–55] and 10 articles included colorectal emergent patients [32–34, 36, 37, 43, 45, 49]– [50] − 6 articles included emergent and elective colorectal patients [32–34, 43, 45] and [51]. None of the articles reported the results separately for colorectal emergent surgery patients.

None of the reviewed articles explored colorectal surgery as a potential risk factor for incisional hernia through univariate or multivariate analyses. Furthermore, none of the articles addressed the usage of parastomal mesh in their analyses.

Only four articles provided separate findings for abdominal emergency patients [37, 47, 49] and [50], while no articles reporting separate findings for emergency colorectal surgery. None of the articles had explored emergency surgery as a potential risk factor for incisional hernia through univariate or multivariate analysis.

### Inclusiveness of colorectal and emergency surgery patients per key question

Risk assessment tools for incisional hernia were assessed in 3 articles [24–26]. Table 4 provides a summary of the key characteristics analysed across the included studies regarding incisional hernia risk assessment and related risk/protective measures per key question (KQ) of the updated guidelines [10]. Twenty four articles assessed risk of incisional hernia as an outcome [18–26, 28, 33, 34, 41–51], 14 articles assessed closure of laparotomy incisions [32–39, 42, 47–51] and 4 articles assessed postoperative care [52–55].

**Table 4.**
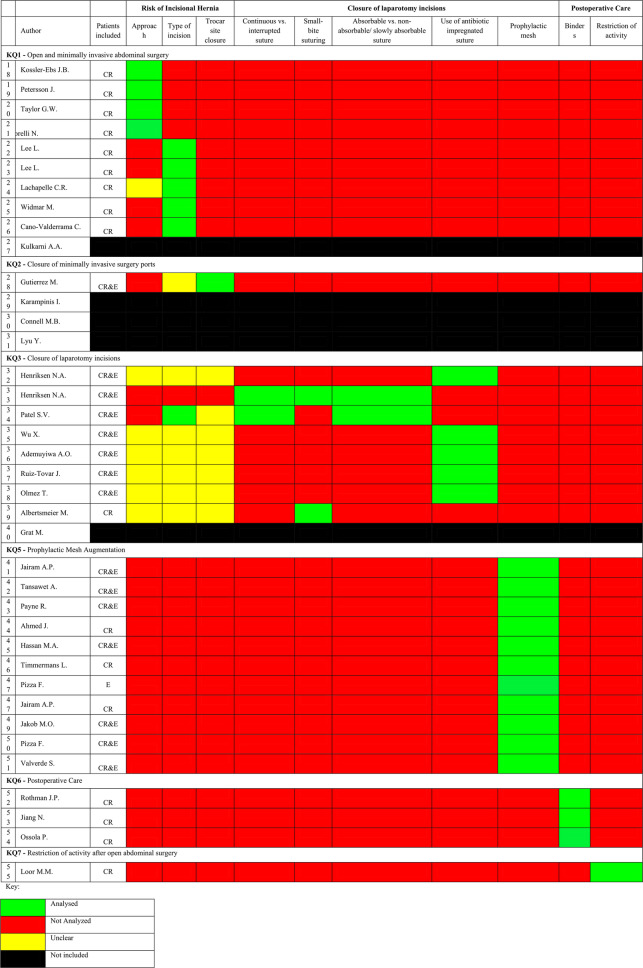
Outcomes: Incisional hernia risk assessment and related risk/protective measures per key question

*CR *included colorectal surgical patients, *E* Included emergency surgical patients, *CR&E* Included colorectal and emergency surgery patients

## Key questions

The recommendations of the updated 2022 EHS/AHS guidelines on abdominal wall closure are structured around seven predefined clinical questions, referred to as “Key Questions” (KQ). Each question addresses a specific aspect of surgical decision-making relevant to the prevention of incisional hernia. Our analysis followed the structure of the guidelines and assessed the inclusiveness and relevance of the cited evidence for colorectal and emergency surgery patients, where applicable. Key Question 4, which focuses on fascial closure in paediatric patients, was excluded from this analysis as considered off topic.

### KQ1 - open and minimally invasive abdominal surgery

Key Question 1 explores whether the type of surgical approach or incision affects the risk of incisional hernia. Of the eleven studies cited in the guidelines, ten were analysed (excluding the original guideline [10]), nine of which included colorectal surgery patients—eight with 100% and one with 64.3% colorectal populations”). - Table 4 [18–26] None of the studies included emergency surgery patients. Four (4/9) studies evaluated the impact of surgical approach on hernia risk [18–21], while five assessed incision type [22–26]. Only two had incisional hernia as a primary outcome, and outcome reporting was heterogeneous in terms of definitions, follow-up, and diagnosis; no study stratified results by urgency or indication.

### KQ2 - closure of minimally invasive surgery ports

Key Question 2 evaluates whether fascial closure of laparoscopic or robotic ports reduces port-site hernia. Four studies were cited [28–31], only one [28] of which included colorectal and emergency patients, without specifying proportions or analysing subgroups. None of the studies provided stratified outcomes or specified closure techniques in detail.

### KQ3 - closure of laparotomy incisions

Key Question 3 investigates which closure techniques and materials are most effective in laparotomy incisions. Of nine studies cited in the guidelines, eight were included in our analysis. All eight involved colorectal and/or emergency patients, although subgroup proportions were not consistently reported [32–39]. One study included only colorectal emergency patients [37], two included predominantly colorectal patients [32, 39], one included >50% emergency patients [36], and three did not specify subgroup proportions [33–35]. Various closure techniques were analysed, including continuous vs. interrupted sutures [33, 34], small-bite technique [33, 39], absorbable vs. non-absorbable sutures [33, 34], and antiseptic (triclosan-coated) sutures [32, 35–38]. No study was industry-funded.

### KQ5 - prophylactic mesh augmentation

Key Question 5 addresses whether prophylactic mesh placement at the time of laparotomy closure reduces the risk of incisional hernia in high-risk patients. All eleven studies cited in the guidelines were included in our analysis [41–51], and all involved colorectal and/or emergency surgery patients. Eight studies reported subgroup proportions [42,43,46,48,51 for colorectal; 47,49–51 for emergency], with two including 100% emergency patients [49, 50]. All studies assessed mesh augmentation; nine detailed mesh positioning, while in two cases it was either unclear or not reported [44, 51]. Four studies were industry-funded [46, 48, 49, 51].While the use of mesh was consistently associated with lower hernia rates, none of the studies stratified this effect by patient type, urgency, or indication.

### KQ6 - postoperative care

Key Question 6 evaluates whether postoperative use of abdominal binders reduces the risk of incisional hernia. Three studies were included [52–54], all of which involved colorectal patients, though only two reported their proportions (6.2% and 34.9%) [53, 54] None of the studies included emergency patients, and outcomes were not stratified by diagnosis or urgency. All studies analysed binder use during postoperative recovery, with heterogeneity in duration, compliance, and definition of hernia.

### KQ7 - restriction of activity after open abdominal surgery

Key Question 7 examines whether limiting physical activity after open abdominal surgery reduces the risk of incisional hernia. Only one study [55] was included in the guidelines under this question. It involved colorectal patients but did not report their proportion, nor did it include emergency patients. The study evaluated outcomes related to early versus delayed return to normal activity but did not stratify results by patient type or indication.

## Discussion

This review highlights the substantial limitations in the current evidence base used to formulate guidelines for abdominal wall closure in colorectal and emergency surgery patients. Although the updated 2022 EHS/AHS guidelines comprehensively summarised available data [10], patients undergoing colorectal or emergency surgery represented only a small fraction of the study populations, limiting the applicability of the recommendations to these high-risk groups. Approximately 15% of the patients in the guidelines’ evidence sources belonged to these populations, yet outcomes were rarely stratified by diagnosis, urgency, or surgical indication, or included specific subgroup analyses. Most studies were underpowered to detect differences among high-risk groups, and incisional hernia was not consistently defined as a primary outcome, further limiting their ability to guide targeted recommendations.

Based on our analysis, recommendations KQ1, KQ3, KQ5 appear relatively reliable for colorectal patients, while in others (KQ2, KQ6, KQ7) evidence is insufficient or extrapolation cannot be made. Key question 1 (“Open and minimally invasive abdominal surgery”) was based largely on evidence from colorectal patients, suggesting reasonable applicability of these recommendations to this subgroup. Colorectal patients were also present in evidence cited for key questions 3 and 5 (“Closure of laparotomy incisions” and “Prophylactic mesh augmentation”), although objective criteria to define the adequacy of this representation were lacking. The exact proportion of colorectal patients could not be ascertained in all systematic reviews and meta-analyses analysed. For key question 6 (“Postoperative care”), while all studies included colorectal patients, the low numbers warrant caution when extrapolating recommendations to clinical practice. In contrast, for key question 2 (“Closure of minimally invasive surgery ports”), colorectal representation was insufficient; however, the clinical impact of this lack of evidence for this group may be negligible. For key question 7 (“Restriction of activity after open abdominal surgery”), colorectal patients were included, but the exact numbers were not specified, impairing assessment of recommendation strength.

Regarding emergency patients, no studies were included for key questions 1, 2, 6, and 7, making extrapolation unjustified, whereas for key questions 3 and 5 the inclusion of emergency patients may support broader applicability.

Of the thirty-three studies, twenty-four included incisional hernia as a primary endpoint, with a median follow-up duration of 24 months. Longer follow-up could reveal higher incisional hernia rates, but failures of technical nature should be expected to occur in the first two years after surgery [56]. Substantial inconsistency was found between studies in how incisional hernia was assessed and reported. Definitions, diagnostic modalities, and observer types varied: some studies used imaging (ultrasound or cross-sectional imaging, which substantially increase detection rates [6]), while others relied solely on clinical examination or even telephone-based patient self-assessment. These inconsistencies likely contributed to outcome heterogeneity and could lead to underreporting (clinical assessment) or misclassification (self-assessment). Moreover, patient-reported outcomes (PROMs), such as pain, satisfaction with body image, and quality of life [57], were rarely evaluated. Future studies should aim to comprehensively capture both objective and patient-centred outcomes to inform more meaningful recommendations.

Systematic reviews were generally rated as low quality, with frequent absence of a pre-registered protocol, incomplete grey literature searches, and inconsistent inclusion of content experts during the literature identification phase. Most systematic reviews searched at least two databases and detailed their search strategies, but risk of bias assessment was often only partially performed or not systematically incorporated into the interpretation of results. Meta-analytic techniques varied widely, and only a minority of reviews assessed publication bias. Among the included randomised controlled trials, risk of bias was mainly due to measurement of the outcome and missing data [58]. Observational studies consistently presented serious risk of bias, primarily because of selection bias. The very low quality of four observational studies was attributed mainly to inconsistency and indirectness, as heterogeneous patient populations hampered generalisability. Only six studies differentiated between emergency and elective surgery, and eleven studies did not report the number of colorectal patients included, further hindering interpretation.

Regarding funding bias, four out of the eleven studies addressing key question 5 reported industry sponsorship [46, 48, 49, 51], with all studies receiving funding from mesh-producing companies, raising concerns about potential bias. Only three studies across the entire dataset evaluated robotic surgery, despite its growing role in colorectal procedures, and none of the studies addressing emergency patients reported on minimally invasive approaches. These limitations demonstrate the overall paucity and low quality of evidence available to inform specific recommendations for colorectal and emergency surgery patients.

### Strengths and limitations

This is the first article critically appraising the evidence supporting the European and American Hernia Society (EHS/AHS) guidelines on abdominal wall closure in colorectal and emergency surgery patients, adding important data for readers, researchers and policymaker and paving the way for further studies on the topic.

For this inherent methodology we focused solely on the sources included in the 2022 EHS/AHS guidelines, without systematically incorporating newly published studies on the topic [7].

We acknowledge that the reported figure of 133,460 patients represents the total number of patients across the studies cited in the EHS/AHS guidelines, and thus may include overlap between systematic reviews and their underlying primary studies. This figure should therefore be interpreted as a representative measure of the evidence base size, rather than the exact number of unique patients. Our conclusions are based on study quality and inclusiveness of colorectal and emergency subgroups, not on the absolute patient count. We added also this statement in the strength and limitations subsection of the study. Furthermore, where full datasets were not available from the articles or supplementary materials, attempts were made to contact the corresponding authors, but noresponses were received. As a result, our ability to reanalyse or verify subgroup-specific outcomes was limited.

### Impact for practice and future research

Key Questions 6 and 7, addressing postoperative care and restriction of activity after open abdominal surgery, highlight important gaps in current evidence. Although the use of abdominal binders has been explored in some studies, including one suggesting potential benefits on physical and mental wellbeing [59], this was not incorporated into the guidelines, and the evidence remains insufficient to support clear recommendations. Moreover, the underlying studies were small, with missing data and suboptimal design, limiting their reliability. Regarding activity restriction, no recommendation could be made due to the complete lack of high-quality evidence, underscoring an urgent need for prospective trials and robust observational studies, ideally based on registries with long-term follow-up and standardised definitions.

Overall, the quality of evidence underpinning the guideline recommendations remains low or very low, and the strength of recommendations was generally weak across all key questions. Within both elective and emergency colorectal surgery, further research is needed to clarify the interrelationship between surgical site occurrences, surgical site infections, and incisional hernia development. While SSI is recognised as a major risk factor for incisional hernia [1, 11, 12], the effect of SSI prevention strategies on the incidence of hernia has not been systematically investigated. Notably, although the updated guidelines report no evidence that antimicrobial-coated sutures prevent incisional hernia, the cited studies only had 30 days of follow-up, sufficient for SSI detection but inadequate for hernia assessment. Longer-term studies evaluating the true impact of SSI prevention measures on hernia formation are therefore necessary.

The results of our analysis underscore that baseline data on specific subgroups in the field of abdominal wall closure are still lacking and the generalization based on extending the results from different procedures could introduce further bias rather than overcome the current dilemmas. In a recent metanalysis including 41 Randomized control trials (RCT) and 9 prospective studies [60], small-bites technique with a slowly absorbable suture showed significantly better results over large-bite technique in elective surgery, while continuous modified Smead-Jones suturing showed a significantly better profile in the emergency setting, suggesting that difference may exist between these populations. For elective patients, the long-term outcome data from STICHT trial [61] are awaited shortly and could add further high-quality data to the current debate, even regarding different subgroups of patients.

Future research should also aim at harmonising abdominal wound closure techniques while developing patient-specific approaches based on individual risk factors. Tailored strategies may be particularly beneficial for high-risk subgroups such as those undergoing emergency colorectal surgery. Furthermore, given the significant impact of both SSI and incisional hernia on quality of life, collecting patient-reported outcome measures (PROMs) is essential to investigate the impact of these complications on patients. Outcomes that are meaningful to patients, including perceptions of surgical wound healing, physical function, cosmetic outcomes, and tolerance of supportive interventions such as binders and exercise restrictions, deserve greater attention.

## Conclusions

Although colorectal and emergency surgery patients were included in the EHS/AHS guidelines, these groups were not sufficiently represented across the evidence base supporting all key questions. Our analysis highlights that not all recommendations can be safely extrapolated to colorectal patients, and even less so to emergency surgery patients. Furthermore, the limited inclusion of patient-centred outcomes, such as quality of life and functional recovery, underscores important gaps in the current literature. There is a clear need for future research specifically addressing the impact of colorectal and emergency surgery on abdominal wound closure techniques and subsequent incisional hernia development. Strengthening the evidence in these areas will enable surgeons to offer patients better-informed counselling and to select closure strategies that are both evidence-based and individually tailored.

## Supplementary Information

Below is the link to the electronic supplementary material.

Supplementary Material 1 (DOCX 2.66 MB)
