# Supplementary material for: Colorectal and emergency surgical patients in the literature supporting EHS and AHS guidelines on abdominal wound closure: a granular analysis
Source: Hernia. 2025 Oct 22;29(1):307. doi: 10.1007/s10029-025-03493-7 (PMC12546287; doi:10.1007/s10029-025-03493-7)

Supplementary files

# Data extraction form

## General Information and article identification

| **1.1** Reference number (*number*)* |  |
| --- | --- |
| **1.2** Reviewer name * |  |
| **1.3** What **main topic** was this article considered for, in the updated guidelines? (you can select more than one option) | Open and minimally invasive abdominal surgery  Closure of minimally invasive surgery ports  Closure of laparotomy incisions  Identifying patients with an increased risk of incisional hernia development  Prophylactic mesh augmentation  Postoperative care |
| **1.4** First author (*surname*) |  |
| **1.5** Year of publication* |  |
| **1.6** Journal/Source (full name) |  |
| **1.7** Study design* | Systematic review/Meta-analysis  Randomised controlled trial  Non randomised or non-controlled trial  Observational study (prospective/retrospective)  Case-control study  Cross-sectional study,  Other (not described) |
| **1.8** Notes/short description/randomisation arms and no randomised per arm (optional) |  |

## *Compulsory

### Participants and Intervention

| **2.1 TOTAL** number of patients included (*number*)* | ___ (Put “0” if none and “000” if not specified) |
| --- | --- |
| **2.2** Did the study include **EMERGENT AND/OR COLORECTAL** surgery patients? | Yes  No  (If “No”, finish data extraction for this article here) |
| **3. EMERGENCY SURGERY** | |
| **3.1** Were abdominal **EMERGENCY SURGERIES** included?* | Yes  No (excluded)  Unclear/Not discriminated  (If “No” or “Unclear/Not discriminated”, go to section “**4.** COLORECTAL SURGERY”) |
| **3.2** Total number of abdominal **EMERGENCY SURGERY** patients included (*number*)* | ___ (Put “0” if none and “000” if not specified) |
| **3.3** Were **OPEN APPROACH EMERGENCY** abdominal **SURGERIES** included?* | Yes  No  Unclear/Not discriminated |
| **3.4** Were **LAPAROSCOPIC EMERGENCY** abdominal **SURGERIES** included?* | Yes  No  Unclear/Not discriminated |
| **3.5** Were **ROBOTIC EMERGENCY SURGERIES** included?* | Yes  No  Unclear/Not discriminated |
| **3.6** Have the findings of **EMERGENCY SURGERIES** been reported SEPARATELY (alone) in the paper?* | Yes  No  Unclear/Not discriminated |
| **3.7** Have the findings of **EMERGENCY COLORECTAL SURGERIES** been reported SEPARATELY (alone) in the paper?* | Yes  No  Unclear/Not discriminated |
| **3.8** Was emergency surgery a **RISK FACTOR** for incisional hernia in univariate and/or multivariate analysis | Not reported/Not applicable  None  Only in univariate analysis  Both in univariate and multivariate analysis |
| **4. COLORECTAL SURGERY** | |
| **4.1** Were abdominal **COLORECTAL** **SURGERIES** included?* | Yes  No (excluded)  Unclear/Not discriminated  (If “No” or “Unclear/Not discriminated”, go to section “**5.** INTERVENTION-GENERAL) |
| **4.2** Total number of abdominal **COLORECTAL** **SURGERY** patients included (*number*)* | ___ (Put “0” if none and “000” if not specified) |
| **4.3** Were abdominal **COLORECTAL ELECTIVE SURGERIES** included?* | Yes  No  Unclear/Not discriminated |
| **4.4** Total number of **COLORECTAL ELECTIVE SURGERY** patients included (*number*)* | ___ (Put “0” if none and “000” if not specified) |
| **4.5** Were abdominal **COLORECTAL EMERGENCY** **SURGERIES** included?* | Yes  No  Unclear/Not discriminated |
| **4.6** Total number of **COLORECTAL EMERGENCY** **SURGERY** patients included (*number*)* | ___ (Put “0” if none and “000” if not specified) |
| **4.7** Were OPEN approach **COLORECTAL** **SURGERIES** included?* | Yes  No  Unclear/Not discriminated |
| **4.8** Were OPEN approach **COLORECTAL EMERGENCY** **SURGERIES** included?* | Yes  No  Unclear/Not discriminated |
| **4.9** Were OPEN approach **COLORECTAL ELECTIVE SURGERIES** included?* | Yes  No  Unclear/Not discriminated |
| **4.10** Were **LAPAROSCOPIC COLORECTAL SURGERIES** included?* | Yes  No  Unclear/Not discriminated |
| **4.11** Were **ROBOTIC COLORECTAL SURGERIES** included?* | Yes  No  Unclear/Not discriminated |
| **4.12** If colorectal surgery was included and discriminated, which procedures were included?  (*you can select more than one option*) | Major resection with primary anastomosis  Major resection with derivative stoma creation  Major resection (no discrimination regarding stoma creation)  Colostomy/Ileostomy creation  Colostomy/Ileostomy closure  Other non specified above (not resection)  Not specified |
| **4.13** Have the findings of **COLORECTAL PATIENTS/SURGERIES** been reported SEPARATELY (alone) in the paper?* | Yes, it was reported separately  Yes, only colorectal patients/surgeries were included in this paper  No  Unclear/Not discriminated |
| **4.14** Was colorectal surgery a risk factor for incisional hernia in univariate and/or multivariate analysis? | Not reported/Not applicable  None  Only in univariate analysis  Both in univariate and multivariate analyses |
| **4.15** Have the findings of **COLORECTAL EMERGENCY SURGERIES** been reported SEPARATELY (alone) in the paper?* | Yes  No  Unclear/Not discriminated |
| **4.16** Have the findings of **COLORECTAL LAPAROSCOPIC SURGERIES** been reported SEPARATELY (alone) in the paper?* | Yes  No  Unclear/Not discriminated |
| **4.17** Was colorectal malignancy included in the surgical indications? | Yes  No  Indications not discriminated |
| **4.18** Has the use of **PROPHYLACTIC PARASTOMAL MESH** been addressed in the paper?* | Yes  No  Not discriminated/ Not applicable |
| **4.19** If the answer to the previous question was “Yes” (prophylactic parastomal mesh was addressed), discriminate mesh position | Not specified in the article  Position: ___________ |
| **5. INTERVENTION - GENERAL** | |
| **5.1** Were risk assessment tools for incisional hernia used in the paper? | Yes  No  Unclear/Not discriminated |
| **5.2** How many study groups/subgroups were analysed or randomised in this paper? | 1  2  ≥3  Not applied |
| **5.3** Was other than midline laparotomy included/discriminated?* | Yes  No  Unclear/Not discriminated |
| **5.4** Are **POSTOPERATIVE COMPLICATIONS/MORBIDITY** addressed in the paper? | Yes  No  Unclear/Not discriminated |

## *Compulsory

###

### 6. Outcomes

|  | | **Description** |
| --- | --- | --- |
| **6.1** Was assessing the **RISK OF INCISIONAL HERNIA** an outcome? *  Yes  No  Unclear | **6.1.1** Was **APPROACH** (open vs minimally invasive) assessed for prediction of incisional hernia risk?* | Yes  No  Unclear/Not discriminated/Not applicable |
|  | **6.1.2** Was **TYPE OF INCISION** (midline vs. off midline) assessed for prediction of incisional hernia risk?* | Yes  No  Unclear/Not discriminated/Not applicable |
|  | **6.1.3** Was **TROCAR SITE CLOSURE** assessed for prediction of incisional hernia risk?* | Yes  No  Unclear/Not discriminated/Not applicable |
| **6.2** Was **CLOSURE OF LAPAROTOMY INCISIONS** assessed? *  Yes  No  Unclear/Not discriminated/Not applicable | **6.2.1** Was **CONTINUOUS vs INTERRUPTED** suture assessed?* | Yes, separately  Yes, but not separately  No  Unclear/Not discriminated/Not applicable |
|  | **6.2.2** Was **SMALL-BITE** suturing assessed?* | Yes, separately  Yes, but not separately  No  Unclear/Not discriminated/Not applicable |
|  | **6.2.3** Was **ABSORBABLE vs. NONABSORBABLE or SLOWLY ABSORBABLE** suture assessed?* | Yes, separately  Yes, but not separately  No  Unclear/Not discriminated/Not applicable |
|  | **6.2.4** Was the use of **ANTIBIOTIC IMPREGNATED SUTURE** assessed?* | Yes, separately  Yes, but not separately  No  Unclear/Not discriminated/Not applicable |
|  | **6.2.5** Was the use of **PROPHYLACTIC MESH** assessed?*  (other than parastomal mesh) | Yes  No  Unclear/Not discriminated/Not applicable |
|  | **6.2.6** If the answer to the previous question was “Yes” (prophylactic mesh was addressed), discriminate mesh position: | Not discriminated  Position: ________ |
| 6.3 **POSTOPERATIVE CARE** | **6.3.1** Was the use of **BINDERS** assessed?* | Yes, separately  Yes, but not separately  No  Unclear/Not discriminated |
|  | **6.3.2** Was **RESTRICTION OF ACTIVITY** assessed?* | Yes, separately  Yes, but not separately  No  Unclear/Not discriminated |
| Median follow-up time *(months)** | | ___ “000” if not specify or not discriminated |

## *Compulsory

ROB-2 Assessment for RCT included in the guidelines


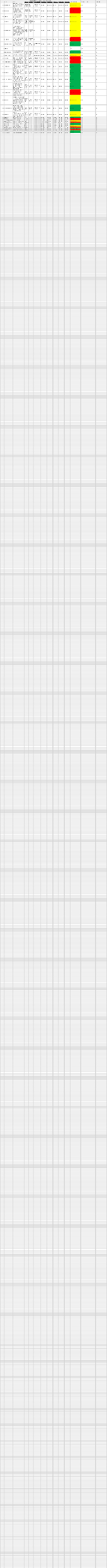


GRADE for the observational studies reported in the guidelines

| **Author** | **Year** | **Design** | **Initial quality of evidence grade** | **Study limitations** | **Inconsistency** | **Indirectness** | **Imprecision** | **Publication bias** | **Magnitude of effect** | **Quality** |
| --- | --- | --- | --- | --- | --- | --- | --- | --- | --- | --- |
| Taylor | 2010 | Retrospective | Low | Retrospective Patient group out of previous RCT Laparoscopic assisted, not totally laparoscopic  High conversion rate to open surgery Selection bias | Relatively low incisional hernia rate | Incisional hernia only assessed clinically and noted |  |  |  | Very low |
| Pecorelli | 2016 | Retrospective | Low | Retrospective Patient group out of previous RCT Prospectively maintained database Single centre | Relatively low incisional hernia rate | Incisional hernia only assessed clinically |  |  |  | Low |
| LaChappelle | 2020 | Retrospective | Low | Retrospective Unknown if patients were consecutive, partly prospective Single centre | Relatively low incisional hernia rate | Incisional hernia assessed clinically or with imaging | Pfannenstiel: high reoperation |  |  | Very low |
| Widmar | 2020 | Retrospective | Low | Retrospective Unknown if patients were consecutive Partly prospective collected data Median follow-up only 14 months with wide interquartile range  Single centre, single surgeon |  | Incisional hernia assessed clinically or with imaging (75%) |  |  |  | Very low |
| Cano-Valderrama | 2020 | Retrospective | Low | Retrospective Uknown if patients were consecutive Single centre |  | Incisional hernia assessed clinically or with imaging if doubt |  |  |  | Very low |

ROBINS-I for the observational studies reported in the guidelines
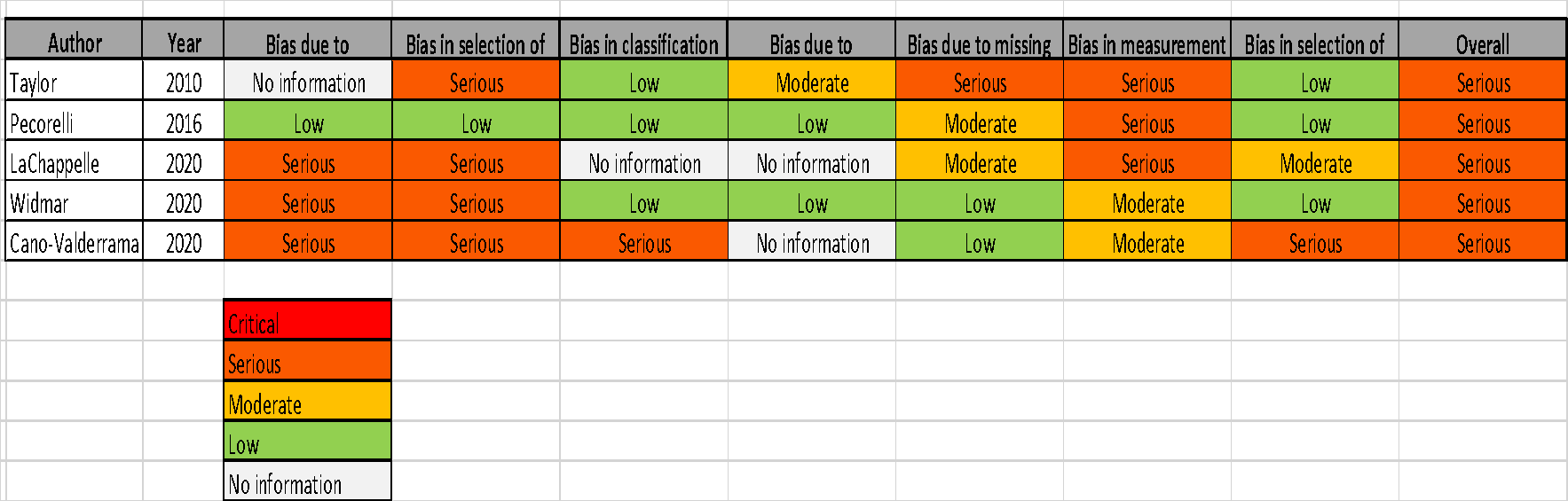

Supplement: Supplementary file 1 — Supplementary Material 1 (DOCX 2.66 MB) [file 10029_2025_3493_MOESM1_ESM.docx]
